# New Vectorial Propulsion System and Trajectory Control Designs for Improved AUV Mission Autonomy

**DOI:** 10.3390/s18041241

**Published:** 2018-04-17

**Authors:** Ivan Masmitja, Julian Gonzalez, Cesar Galarza, Spartacus Gomariz, Jacopo Aguzzi, Joaquin del Rio

**Affiliations:** 1 SARTI research group, Electronics Department, Universitat Politècnica de Catalunya, 08800 Vilanova i la Geltrú, Spain; joticajulian@gmail.com (J.G.); cesarmgb@gmail.com (C.G.); spartacus.gomariz@upc.edu (S.G.); joaquin.del.rio@upc.edu (J.d.R.); 2 Marine Science Institute (ICM), Consejo Superior de Investigaciones Científica (CSIC), 08003 Barcelona, Spain; jaguzzi@icm.csic.es

**Keywords:** propulsion system, AUV, autonomous vehicle, linear control, fuzzy control, automatic navigation, thruster vectorial control

## Abstract

Autonomous Underwater Vehicles (AUV) are proving to be a promising platform design for multidisciplinary autonomous operability with a wide range of applications in marine ecology and geoscience. Here, two novel contributions towards increasing the autonomous navigation capability of a new AUV prototype (the Guanay II) as a mix between a propelled vehicle and a glider are presented. Firstly, a vectorial propulsion system has been designed to provide full vehicle maneuverability in both horizontal and vertical planes. Furthermore, two controllers have been designed, based on fuzzy controls, to provide the vehicle with autonomous navigation capabilities. Due to the decoupled system propriety, the controllers in the horizontal plane have been designed separately from the vertical plane. This class of non-linear controllers has been used to interpret linguistic laws into different zones of functionality. This method provided good performance, used as interpolation between different rules or linear controls. Both improvements have been validated through simulations and field tests, displaying good performance results. Finally, the conclusion of this work is that the Guanay II AUV has a solid controller to perform autonomous navigation and carry out vertical immersions.

## 1. Introduction

The ocean interior is invisible to the human eye and comprehension is poor due to the limited range of light, making deep-sea operations inaccessible to humans. With respect to the importance of the oceans on Earth, their habitat and communities contained therein are significantly under-surveyed in time and space [1]. Consequently, our perception of marine ecosystems is fragmented and incomplete. Scattered vessel-assisted sampling methodologies (i.e., ROVs, AUVs or more classic trawling) do not allow changes in communities’ composition (and hence detectable biodiversity) upon species behavior and their spatio-temporal modulation (i.e., under the form of massive and rhythmic populational displacements [2,3]). Robotic platform developments are therefore increasingly pursued to increase the autonomy of monitoring marine environments and their biological components [4,5,6,7].

While autonomy in space and terrestrial robots has seen a significant increase in technological research and derived applications, the underwater domain is still mostly operating in a teleoperated manner. In this development, Autonomous Underwater Vehicles (AUV) are proving to be a promising design for multidisciplinary autonomous operability [8] with a wide range of applications in marine ecology and geoscience [9]. Autonomous navigation capability through depths and over and within complex seabed morphologies is a critical aspect for successful missions, but the absence of an underwater global positioning system (GPS) has forced the development of acoustic modem communicability [10,11].

**Figure 1:**
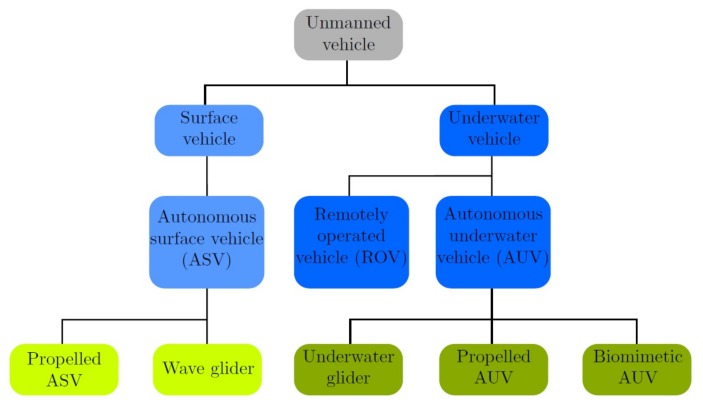
Underwater vehicles’ classification.

Over the last few years, different autonomous vehicles have been developed to cover all the necessities and requirements for underwater research [12], which can be grouped into different types of classifications. For example, one can try to classify vehicles that have the same type of power supply as in [13]. However, an interesting method for AUV classification is through the propulsion method, which influences the design of the model and navigation controls most; see Figure 1.

A particular case of AUV is the AUV glider [14] (p. 407), which uses small changes in its buoyancy in conjunction with wings to convert vertical motion to horizontal motion. While this method is unsuitable for high maneuverability scenarios, it significantly increases the range and duration of its operations, which can be extended from hours to weeks or months, due to its low power consumption.

Other studies have focused on biomimetic AUVs, which copy propulsion systems directly from the animal world [15]. However, the most common and extended propulsion system is through propellers. These vehicles use thrusters that realize their movement due to possessing high maneuverability capabilities and velocity. Therefore, they are usually used for inspection, underwater mapping or intervention [16].

In general, two methods can be found related to vectorial propulsion navigation systems. The first one uses fixed thrusters oriented over the vertical plane. This method is the most common and can be found in a various vehicles, such as [17]. The second method uses a system that can change the angle of the propulsion vector. Some studies have focused on a single thruster design, which can be oriented in different directions, where a joint development between MBARI (Monterey Bay Aquarium Research Institute) and Bluefin Robotics [18] has to be highlighted in this area, among others studies that have used this idea, such as [19]. On the other hand, some studies have used different orientable thrusters, increasing the AUV’s maneuverability (e.g., [20]).

This paper presents the development and improvement of an AUV, the Guanay II, which is a mix between a glider and a propelled vehicle, especially in the area of its navigation control. Most of the state of the art works [21,22,23] design the controllers referencing the hydrodynamic model of the vehicle when it travels at a specific forward velocity in order to simplify the control design. While this is true for a vehicle that habitually navigates in open sea, variations in velocity can be significant in areas near the coast, on the sea floor or in the interior of ports and canals.

Some of the works that propose a solution to this problem use techniques based on Lyapunov functions [24,25]. These solutions lead to a loss of simplicity of the control laws. Moreover, these non-linear techniques do not enjoy the same diffusion and popularity as their linear counterparts.

An alternative is the work of Silvestre and Pascoal [26], where they design linear controllers for different forward velocities and thereafter use a gain scheduling controller to integrate them. Other works focused on using the advantages of gain scheduling controllers, but applying a fuzzy framework to manage them (e.g., [27,28]). However, they use a linguistic interpretation to calculate the parameters of the controller, rather than an analytic procedure.

Finally, in [29], a comparison of the fuzzy controller technique in regard to gain scheduling is presented. They show that fuzzy controllers have similar performances when compared to gain scheduling ones. Thus, because fuzzy controllers perform well, in this work, the use of the type TSK in order to manage different linear controllers designed for specific conditions of forward velocity is proposed, where its analytic development has also been studied. This approach has also been used recently in others papers (e.g., [30]), where the navigation performance was simulated with 6 DOF. However, real field tests are also presented here.

This work, therefore, establishes innovations at the level of hardware and software navigation, to potentiate AUV autonomous operability, by adding novel vectorial propulsion insight to across-depth navigation and trajectory control. Vectorial propulsion systems are widely used, especially in Remotely-Operated Vehicles (ROVs). However, in AUVs, those methods are, comparatively, less implemented. From a trajectory control systems design point of view, advances in the use of methods for motion control that rely heavily on fuzzy techniques are presented.

The following sections are structured as follows: the main architecture of Guanay II is described in Section 2, where the horizontal and vertical navigation and propulsion systems are presented; Section 3 develops the inner and outer loops, describing the control of the thrusters and navigation capabilities in the horizontal plane; finally, in Section 4, the results are presented, the outcomes of both simulations and field test trials. To conclude, discussions and conclusions are presented in Section 5 and Section 6, consecutively.

## 2. Materials and Methods

### 2.1. Guanay II AUV Architecture

**Figure 2:**
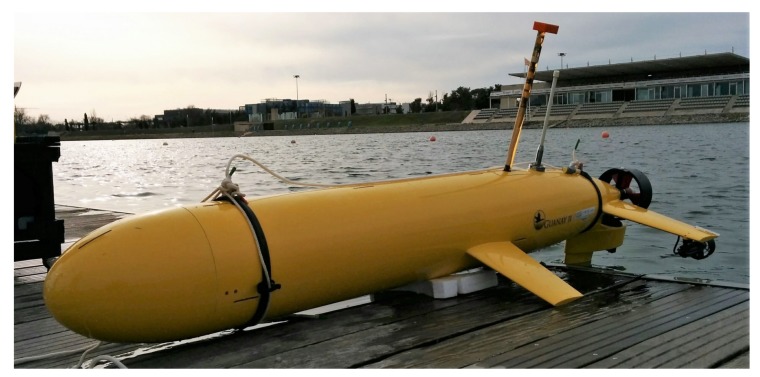
The Guanay II AUV [31] docked at the SARTI (Universitat Politecnica de Catalunya (UPC)) harbor facilities. Image taken during field tests at the Olympic Canal in Castelldefels.

The Guanay II AUV (Figure 2) is a vehicle under permanent development constructed by SARTI Research group (www.cdsarti.org) from the Universitat Politecnica de Catalunya (UPC, www.upc.edu). This vehicle was initially designed to perform water-column measurements. With high surface stability through its fin stabilizers mounted in the hull, the Guanay II can navigate on the sea surface and perform vertical immersions to take measurements of different water parameters, such as Conductivity, Temperature and Depth (CTD). The immersion system consists of a piston-engine mechanism varying the buoyancy according to a remotely-enforced schedule (see below), which can take 1.5 L of sea water. The propulsion system consists of one main 300-W nominal power thruster (a Seaeye SI-MCT01-B) and two smaller lateral thrusters to control the direction of the vehicle (Seabotix BTD150). These devices are controlled by an on-board embedded computer and communicated with radio frequency modems to the user station.

Manufactured with fiberglass, the hull of Guanay II was designed to give the maximum stability in horizontal navigation. Taking into account that the main part of the vehicle’s mission is navigating on the sea surface, the Guanay II incorporates different fins to give the necessary stability to navigate through the waves. This is an important difference with respect to other AUV, which are designed to navigate mostly underwater.

On the other hand, to maximize the efficiency, the hull follows the Myring profile [32], which allows good performance in navigation through the water due to its low drag coefficient. Moreover, different blocks of foam can be added to obtain the desired flotation, and by moving the position of the ballast system, located at the bottom, the attitude can be adjusted.

### 2.2. Mathematical Model for Autonomous Navigation

A mathematical model for the autonomous navigation capability of Guanay II AUV has been elaborated in order to simulate the performance of the vehicle in open loop and to design controllers. However, modeling a marine vehicle, which is moving inside a turbulent fluid, is a complex task. In general, one can encounter two main difficulties: the selection of the coefficients and secondly their calculation. Different studies have been done to solve these problems [33,34,35].

**Figure 3:**
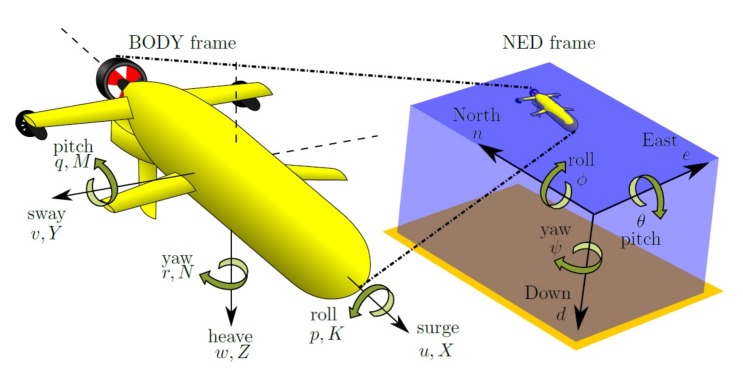
Body frame and NED frame representation of linear velocities [uvw], forces [XYZ], angular velocities [pqr], attitude [ϕθψ] and torque [KMN].

The forces and torques that generate the vehicle’s accelerations are represented in an equation, which is given as a function of the velocity vector v. Figure 3 shows the vehicle coordinates, velocities and forces. Moreover, the rigid body dynamics must take into account the Coriolis and centrifugal effects. For simplicity, they are usually calculated in the body frame. Using all of these considerations, the dynamics of the vehicle can be described as follows, as is proposed in [33].

(1) $$\left(M_{RB}+M_{A}\right)\dot{v}+\left(C_{RB}+C_{A}\right)v+D_{n}v=\tau ,$$

where $M_{RB}$ is the rigid body inertia matrix, $C_{RB}$ is the rigid body Coriolis and centripetal matrix, $M_{A}$ and $C_{A}$ represent the added mass matrices and $D_{n}$ represent the sum of non-linear damping factors. Finally, τ represents the control input vector. All of these matrices depend on several coefficients, whose strict definition is the partial derivative of a force or torque that actuates in the vehicle with respect to a velocity or an acceleration and is evaluated at the origin. These parameters can be derived mathematically or through field experiments, but no standard method is used.

### 2.3. Horizontal Navigation and Propulsion System

In order to simplify the model, the Six Degrees Of Freedom (DOF) model can be uncoupled into a 3 DOF, where the Guanay II AUV moves only on the surface: surge, sway and yaw. The movement in the other coordinates can be disregarded. This method, known as the divide and conquer strategy, is a common strategy in these types of problems. In this situation, the velocity vector becomes $v={\left[u\;v\;r\right]}^{T}$, where *u* and *v* represent the body-fixed linear velocity on the *x*-axis (surge) and *y*-axis (sway), respectively, and *r* represents the body-fixed angular velocity on the *z*-axis (yaw).

Using this configuration, the control inputs τ consist of a force for surge movement using the three thruster, and a torque for yaw movement using the lateral thrusters as follows:

(2) $$\tau =\left[\begin{array}{c} Prop_{x}\\ 0\\ Torque \end{array}\right]\begin{array}{c} Prop_{x}=X_{main}+X_{lft}+X_{rgt}\\ Torque=a_{fin}\left(X_{lft}-X_{rgt}\right) \end{array},$$

where $a_{fin}$ is the distance from the lateral thrusters to the central axis and $X_{main}$, $X_{lft}$ and $X_{rgt}$ are the forces of the main, left and right thrusters, respectively. All these parameters, which will introduce boundaries on the vehicle’s performance, have to be considered when designing the navigation control (e.g., the maximum velocity or the breaking speed, as well as its turn radius).

### 2.4. Vertical Navigation and Propulsion System

Vertical immersion capability is ensured by an engine-piston set, able to collect and eject 1.5 L of water, which means that it modifies 1.5 kg of Guanay II’s density [36]. Although this system has a slow dynamic behavior, typically tens of seconds, it has a very low power consumption, where energy is used only at the beginning and end of the immersion. Moreover, because no thrusters are used, this method causes low turbulence. Therefore, it is very useful for low power consumption vehicles, such as gliders, and to perform water-column measurements, where no mixing between layers is desired.

Nevertheless, a new thruster vector control system has been designed and implemented to increase Guanay II’s performance and usability. This system can be used to navigate the vehicle during an immersion, as in [37]. The proposed system consists of adjusting the angle between the lateral thrusters and the hull through actuators. Consequently, the pitch angle of the vehicle can be controlled with this modification.

**Figure 4:**
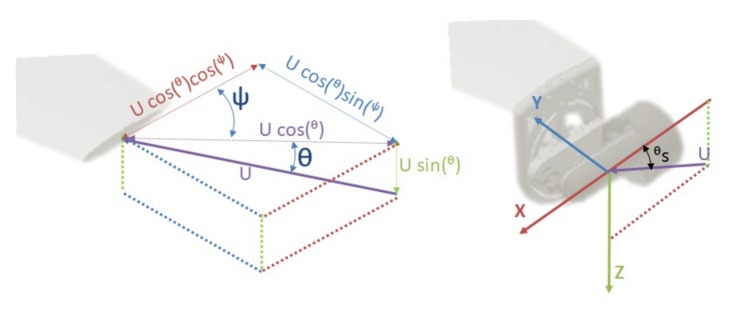
Vector decomposition of the lateral propulsion vector (**left**) and the propulsion vector of the lateral thrusters of Guanay II AUV (**right**).

Similarly as before, the 3 DOF simplified model in the vertical plane can be derived, where the velocity vector state $v={\left[u\;w\;q\right]}^{T}$ represents the body-fixed linear velocities on the *x*-axis (surge) and *z*-axis (heave) and the body-fixed angular velocity on the *y*-axis (pitch), respectively. These velocities are controlled by the input τ, which contains a force for surge movement using the three thrusters, and a torque for pitch movement and heave movement using the lateral thrusters. However, the force of the lateral thrusters has to be decomposed on its *x*-axis and *z*-axis due to their rotation movement (Figure 4). Using this vector movement, the control input τ is defined as:

(3) $$\tau =\left[\begin{array}{c} Prop_{x}\\ Prop_{z}\\ Torque \end{array}\right]\begin{array}{c} Prop_{x}=X_{main}+\left[X_{lft}+X_{rgt}\right]\cos\left(\theta \right)\\ Prop_{z}=\left[X_{lft}+X_{rgt}\right]\sin\left(\theta \right)\\ Torque=a_{cb}\left[X_{lft}+X_{rgt}\right]\sin\left(\theta \right) \end{array},$$

where θ is the angle of the lateral thrusters and $a_{cb}$ is the distance between the lateral thrusters and the center of buoyancy of the vehicle.

**Figure 5:**
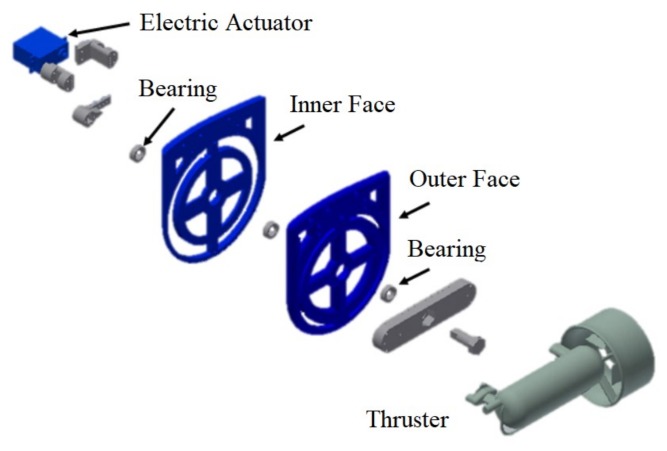
Parts of the structure designed to obtain a thruster vector control on the vertical plane.

The rotation movement is provided by an electric actuator, which is coupled to the thruster through a mechanical frame (Figure 5). This new mechanism is capable of providing ±25 degrees of movement on the Guanay II AUV’s lateral thrusters.

With this new implementation, Guanay II now has full maneuverability, which allows us to control the vehicle in the horizontal and vertical plane.

## 3. Automatic Navigation Control

Here, only the navigation control in the horizontal plane has been addressed, in order to reduce the mathematical complexity.

### 3.1. Autonomous Navigation Development Blocks

**Figure 6:**
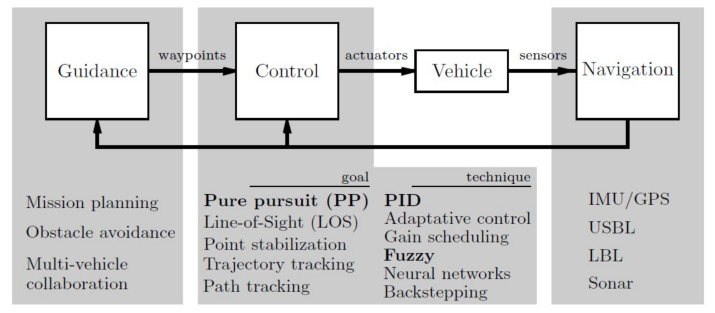
Guidance, navigation and control and the some of the main associated research lines. In bold, those aspects described in greater details in the paper are reported. USBL, Ultra-Short Baseline; LBL, Long Baseline.

In general, the autonomous navigation system is divided into three main layers or subsystems, as can be observed in [38,39], where they present the concept of Guidance, Navigation and Control systems (GNC); see Figure 6.

- Guidance system: This is the highest control level of the vehicle during a mission. Usually, it has a waypoint generator, which establishes the points to cross to accomplish the goal of the mission. Moreover, it can incorporate several algorithms such as path planning, obstacle avoidance or multi-vehicle collaboration, to improve autonomous maneuverability.
- Navigation system: This system receives the sensor’s data, used to compute the vehicle’s position, velocity and linear and angular accelerations. Due to the complexity of the underwater environment, different methods are used, among which the most common are: acoustic systems, such as Ultra-Short Baseline (USBL) and Long Baseline (LBL); dead-reckoning such as Doppler Velocity Log (DVL) and Inertial Measurement Units (IMU).
- Control system: Finally, this system processes information to infer the current state of the vehicle and generate an appropriate command to the actuators to reduce the differences between the actual and desired trajectories.

The control system allows user selection of the waypoints as a function of the mission’s goal. Moreover, to reduce the complexity of the control, the design in the horizontal plane has been selected. This strategy is used because in general, most of the missions are carried out at a constant depth. Therefore, the horizontal and vertical controls can be separated without any loss in their performance, as it is a decoupled system. Finally, it has to be considered that Guanay II was designed to navigate on the sea surface; therefore, the main navigation system in this scenario is the GPS. However, this development can be used for immersed navigation, where coordination between GPS and other underwater navigation systems is required.

In situations such as navigation in harbors or canals, the variation of the forward velocity becomes relevant, and so, it is important to be able to vary the controller’s working point to adjust the paths to the desired ones. To solve this problem, Silvestre and Pascoal [26] use a set of linear controllers adjusted for different forward velocities and then use a gain scheduling controller to integrate them. Here, the same methodology has been followed, but a fuzzy controller has been applied to integrate the different linear controllers. The fuzzy controller allows activation zones to be established, which can be controlled through fuzzy sets. This method, used as interpolation between different rules or linear controls, performs well. In this work, the Takagi and Sugeno (TSK) [40] controller is used.

Finally, the control system has been divided into two loops: the inner loop and the outer loop. The first loop was used for setting the yaw ψ and the forward velocity *u*, given a reference $\left(\psi_{ref},v_{ref}\right)$, and the second loop is responsible for setting the reference for yaw and forward velocity for a given path.

### 3.2. Inner Loop

A set of linear controls is zonally differentiated, if it can be shown that each control is more optimal than the others in a specific zone. For marine vehicles, these zones represent the different forward velocities *u* for which the model is linearized. In this section, a set of zonally-differentiated controls will be developed to control the vehicle yaw angle at different forward velocities.

#### 3.2.1. Linear Controllers

The first step towards designing a linear control is the transfer function of the system, for both the yaw and forward velocity of the vehicle.

For the transfer function of the yaw, the angle ψ can be calculated with the angular velocity *r* through a rotation matrix to change from the body to the North East Down (NED) frame using the Euler angles. On the other hand, the mathematical model of the vehicle defines the relation between the angular velocity and the torque applied by the thrusters. Here, the mathematical model development is not presented, which is presented in [35]. Therefore, only the final equation is used. Finally, by applying the Laplace transform, the following equation, describing the transfer function of the yaw with respect to the torque, can be obtained.

(4) $$\begin{array}{c} G_{\psi }\left(s\right)\\ u_{0} \end{array}=\frac{\psi (s)}{Torque(s)}=\frac{m_{u}s-Y_{u}}{As^{3}+Bs^{2}+Cs},$$

where the sub-index $u_{0}$ represents the velocity at which the model is linearized.

**Figure 7:**
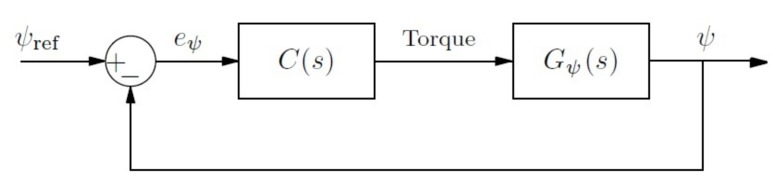
Block diagram of the closed loop system for the yaw control.

Concerning the second aspect, the zonally-differentiated controllers for yaw, the main idea of the control is to bring the error between a reference $\psi_{ref}$ and its present value ψ to zero, using a controller that actuates on the lateral thrusters. The general block diagram can be observed in Figure 7.

Therefore, the transfer function of the yaw with respect to the reference, $H_{\psi }\left(s\right)$, can be calculated as:

(5) $$H_{\psi }\left(s\right)=\frac{\psi (s)}{\psi_{ref}\left(s\right)}=\frac{C\left(s\right)G_{\psi }\left(s\right)}{1+C\left(s\right)G_{\psi }\left(s\right)}.$$

After different simulations [35], it was concluded that the optimal controllers for 0.3 m/s and 2 m/s were: a Proportional-Derivative (PD) control for 0.3 m/s and a Proportional (P) control for 2 m/s, $C_{PD0.3}\left(s\right)$ and $C_{P2.0}\left(s\right)$, respectively. These controllers have the following equation:

(6) $$\begin{array}{c} C_{P}\left(s\right)\\ u_{0} \end{array}=k,$$

(7) $$\begin{array}{c} C_{PD}\left(s\right)\\ u_{0} \end{array}=k_{d}s+d_{p}.$$

Solving these controllers with the transfer function $H_{\psi }\left(s\right)$ and the linearization velocities, the following equations have been obtained:

(8) $$\begin{array}{c} C_{P}\left(s\right)\\ 2.0 \end{array}=1043.8723,$$

(9) $$\begin{array}{c} C_{PD}\left(s\right)\\ 0.3 \end{array}=408.8266\left(s+0.8039\right).$$

**Figure 8:**
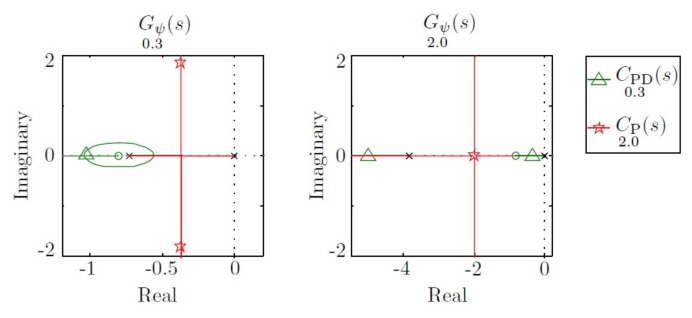
Root locus for $G_{\psi 0.3}\left(s\right)$ and $G_{\psi 2.0}\left(s\right)$ and pole displacement using controllers $C_{PD0.3}\left(s\right)$ and $C_{P2.0}\left(s\right)$.

When the vehicle travels at 0.3 m/s, $C_{PD0.3}\left(s\right)$ moves the two poles to −1, whereas $C_{P2.0}\left(s\right)$ moves them to −0.4±1.8i. In this case, the benefits of the first controller are clear. However, when the vehicle travels at 2 m/s, the first controller moves the dominant pole to −0.3, and the second controller moves the two poles to −1. In this case, the benefits are observed on the second controller. This behavior can be observed in Figure 8.

**Figure 9:**
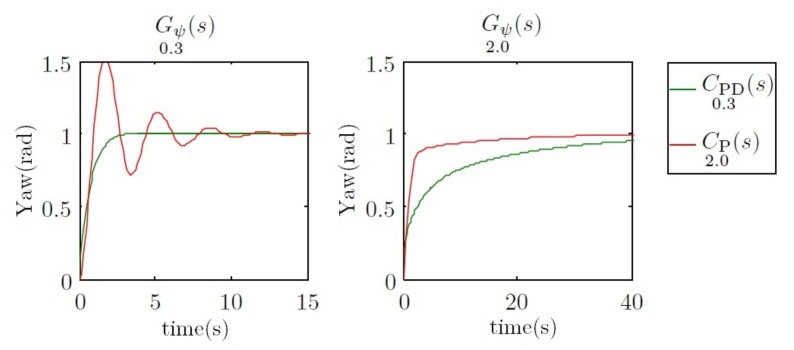
Step response of the systems $G_{\psi 0.3}\left(s\right)$ and $G_{\psi 2.0}\left(s\right)$ using the controllers $C_{PD0.3}\left(s\right)$ and $C_{P2.0}\left(s\right)$ in a feedback loop. The two time scales are different for a better response appreciation between controllers at different velocities.

On the other hand, the step response of both controllers can be observed. In this case, for the first linearization, the $C_{PD0.3}\left(s\right)$ controller yields a better performance than the $C_{P2.0}\left(s\right)$ controller, which has an underdamped and slow response. Nevertheless, this performance is inverted in the second linearization, where the $C_{P2.0}\left(s\right)$ has the fastest response, as can be observed in Figure 9. To conclude, one can say that these sets of controllers are zonally differentiated.

Then, the transfer function of the forward velocity has also been obtained with the vehicle’s mathematical model. The final expression (whose intermediates have already been described in [35]) can be expressed as:

(10) $$\begin{array}{c} G_{u}\left(s\right)\\ u_{0} \end{array}=\frac{u(s)}{Prop(s)}=\frac{1}{m_{u}s-2\left|u_{0}\right|X_{|u|u}-X_{u}},$$

where the sub-index $u_{0}$ represents the velocity at which the model is linearized. On this transfer function, it can be observed that there is only one pole, as a main difference with respect to the previous one.

**Figure 10:**
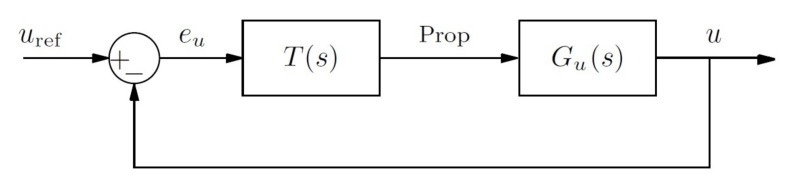
Block diagram of the closed loop system for the velocity control.

Finally, for the forward velocity control, the controller T(s) actuates on the main thruster to bring the error between the reference velocity, $u_{ref}$, and the actual velocity, *u*, to zero. The block diagram is shown in Figure 10.

The transfer function of the velocity with respect to the reference, denoted by $H_{u}\left(s\right)$, can be calculated as:

(11) $$H_{u}\left(s\right)=\frac{u(s)}{u_{ref}\left(s\right)}=\frac{T\left(s\right)G_{u}\left(s\right)}{1+T\left(s\right)G_{u}\left(s\right)}.$$

In this case, the Proportional-Integral (PI) controller is a good option to guarantee zero error in the steady state for a step response. The PI adds an integrator, which implies a root locus with two poles and one zero, which has the following notation:

(12) $$\begin{array}{c} T_{PI}\left(s\right)\\ u_{0} \end{array}=\frac{k_{p}s+k_{i}}{s}.$$

This structure is similar to the disposition obtained in the yaw control using a PD controller. Therefore, a similar design process has been used, linearizing two controllers at 0.3 m/s and 2 m/s respectively, obtaining:

(13) $$\begin{array}{c} T_{PI}\left(s\right)\\ 0.3 \end{array}=\frac{337.6657(s+0.2200)}{s},$$

(14) $$\begin{array}{c} T_{PI}\left(s\right)\\ 2.0 \end{array}=\frac{327.7492(s+1.0820)}{s}.$$

**Figure 11:**
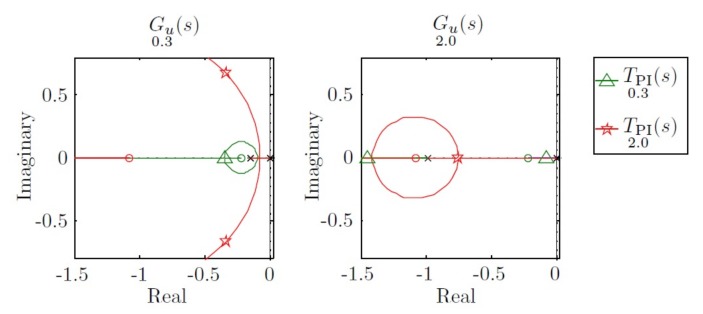
Root locus for $G_{u0.3}\left(s\right)$ and $G_{u2.0}\left(s\right)$ and pole displacement using controllers $T_{PI0.3}\left(s\right)$ and $T_{PI2.0}\left(s\right)$.

At minimum speed, the $T_{PI0.3}\left(s\right)$ moves the poles to −0.35, whereas the $T_{PI2.0}\left(s\right)$ moves the poles to −0.34±0.68i. The real poles represent a better option than the conjugated poles. On the other hand, at maximum speed, the $T_{PI0.3}\left(s\right)$) moves the domain pole to −0.08, which is very near the imaginary axis, and the $T_{PI2.0}\left(s\right)$ moves the poles to −1.4, which guarantees a fast response. This behavior can be observed in Figure 11.

**Figure 12:**
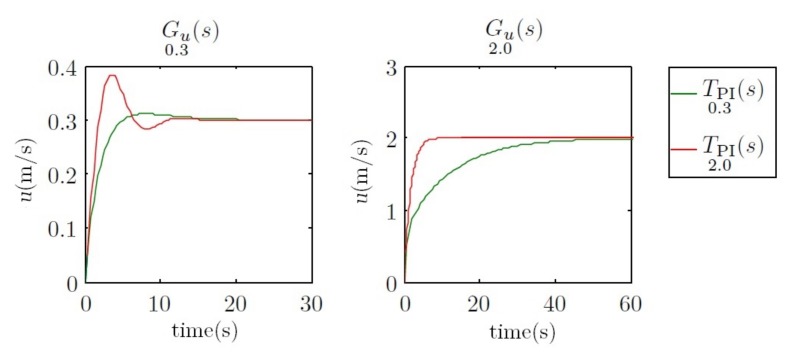
Step response of the systems $G_{u0.3}\left(s\right)$ and $G_{u2.0}\left(s\right)$ using the controllers $T_{PI0.3}\left(s\right)$ and $T_{PI2.0}\left(s\right)$ in a feedback loop. The two time scales are different for a better response appreciation between controllers at different velocities.

Finally, if one looks at the step response of these controllers, a similar performance to that obtained on the yaw control will be observed. In Figure 12, the first control has a faster response with a small overshoot for 0.3 m/s, but a worse response at 2 m/s. The second control has the opposite performance.

#### 3.2.2. Fuzzy Controller

**Figure 13:**
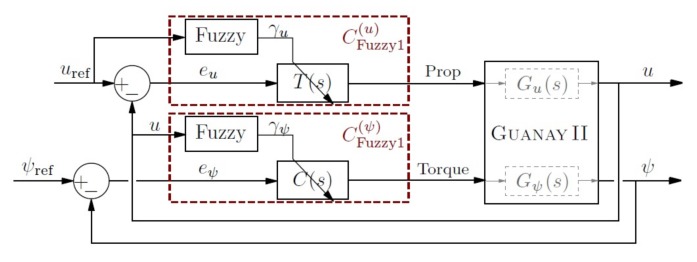
Fuzzy control: velocity and yaw control regarding the forward velocity *u*.

The section above has shown the importance of using different controllers to control the velocity and yaw of the vehicle, depending on its forward velocity. The fuzzy control is a good method to perform this action, which can be used as an interpolating controller using a reasoning rule base to estimate the required control signal. In this paper, two types of controllers are presented, $C_{Fuzzy1}^{\left(u\right)}$ and $C_{Fuzzy1}^{\left(\psi \right)}$, as is shown in Figure 13.

The first controller $C_{Fuzzy1}^{\left(u\right)}$ is used to control the propulsion of the vehicle to reach the desired velocity. In this case, the parameters of linear control T(s) are dynamically modified by a fuzzy block, using the two zonally-differentiated linearizations at 0.3 m/s and 2 m/s.

The second controller $C_{Fuzzy1}^{\left(\psi \right)}$ is used to modulate the torque of the vehicle, to reach the correct yaw. Similarly, the parameters of C(s) are dynamically modified by a fuzzy block, also using also the two zonally-differentiated linearizations at 0.3 m/s and 2 m/s.

**Figure 14:**
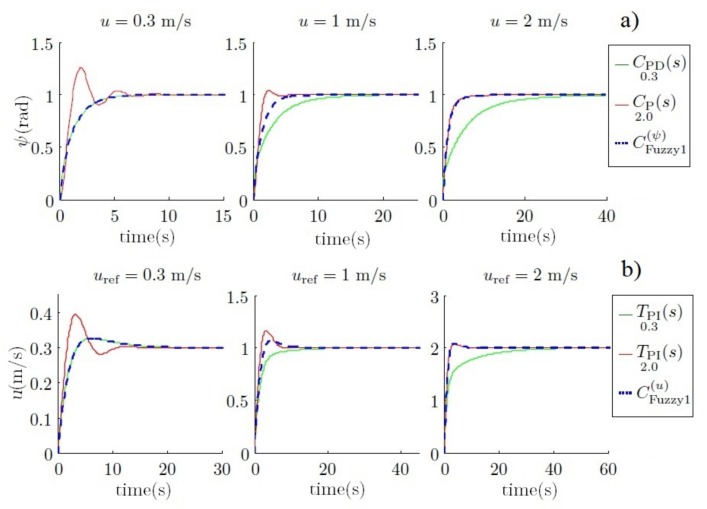
Comparison of the step response for the yaw (**a**) and forward velocity (**b**) using the fuzzy controller (discontinuous blue line) and linear controllers (green and red lines). The time scales are different for a better response appreciation between controllers at different velocities.

Some advantages of using these controllers were presented in previous work [41] and can be observed in Figure 14a for yaw control and in Figure 14b for velocity control. The fuzzy control combines the good performance of both controllers at low and high velocities, which yields a fast and small overshot response, as can be observed.

However, in these simulations, the constraints of the vehicle have not been taken into account, especially for yaw control. For example, to accomplish the turn in the simulated time, torque values up to 3000 Nm would be necessary, and these are impossible to reach with our present mechanical assets.

#### 3.2.3. Constraint Problems for Yaw Control

Guanay II has two main constraints: a torque limit of 28 Nm and an attitude sensing noise of ±5.84 degrees. The torque limit is due to the thrusters used to change the yaw and its position in the vehicle. The attitude noise, which must be considered, is mostly due to the waves, when the vehicle is navigating on the sea surface.

**Figure 15:**
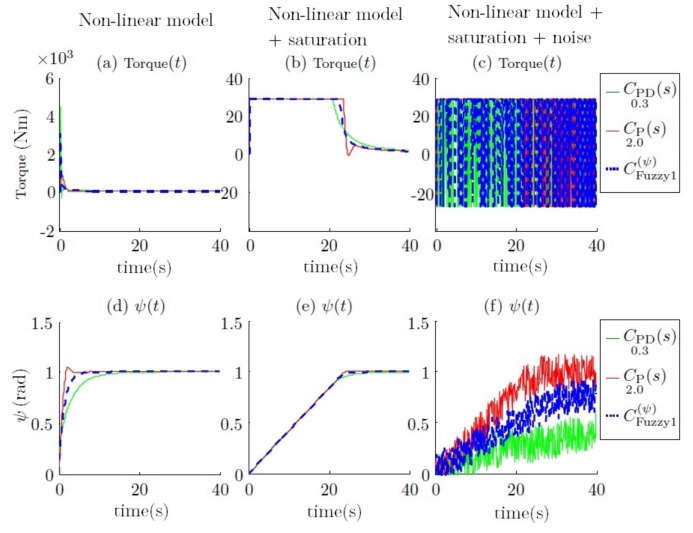
Torque comparison of the step response for the yaw using different controllers when the vehicle is navigating at 1 m/s. Subplots (**a**,**d**) represent the torque and yaw response without constraints. Subplots (**b**,**e**) show the response with physical constraints. Finally, subplots (**c**,**f**) show the response with physical and noise constraints.

**Figure 16:**
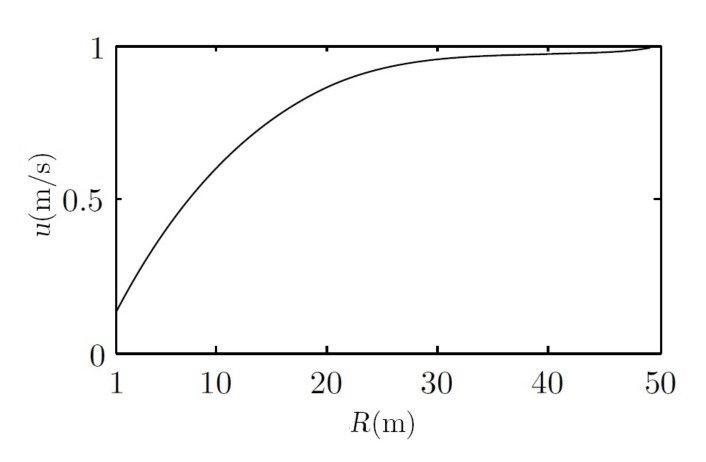
Maximum forward velocity *u* regarding the radius of curvature.

Two main drawbacks are observed when these constraints are introduced into the simulations. The first one is when the torque limit is added; see Figure 15b,e. This constraint is derived from the physical characteristics of Guanay II, which has been taken into consideration in its mathematical model. Moreover, its performance has also been studied in field tests conducted previously [42]. The relationship between forward velocity and turn radius is shown in Figure 16. The main advantage of using a vectorial propulsion system is that the vehicle is able to turn over on its own axis when the forward velocity is low. On the other hand, if higher velocities are desired, the turn radius must increase. Consequently, in this case, the time needed to reach the desired yaw is lower, and no differentiation between controllers is appreciated. Therefore, the advantages of a fuzzy controller are not observed.

The second drawback is when the yaw noise is added (see Figure 15c,f), where the torque required is alternatively saturated in both ways. This means that the thrusters will be continuously switching between their maximum and minimum, producing obvious damage. This problem is due to the derivative action of the controller, which amplifies the noise.

**Figure 17:**
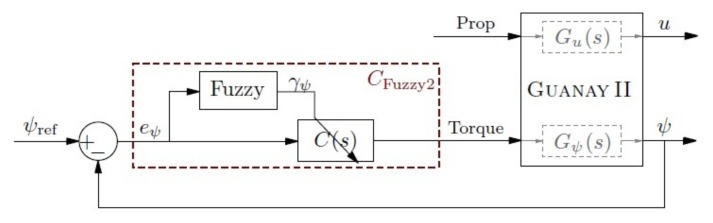
Fuzzy control regarding the yaw error.

Due to these problems, another approach to control the yaw is proposed, $C_{Fuzzy2}$, which consists of using the yaw error as the input of the fuzzy controller instead of the velocity. Its block diagram is shown in Figure 17.

The idea is to use a proportional controller with big gain when the error is big and a small gain when the error is small. With this action, the controller will saturate only with large yaw errors, and not with small yaw errors, avoiding the fast switching. Considering the noise value of ±5.84 degrees (or ±0.102 rad) and the maximum torque for yaw errors of $2^{\circ }$, $10^{\circ }$ and $20^{\circ }$ equal to 2.8 Nm, 4.2 Nm and 11.2 Nm, the following proportional controllers are obtained:

(15) $$C\left(s\right)=k_{p1}=\frac{Torque}{e_{\psi }}=\frac{2.8}{0.102}=27.45,$$

(16) $$C\left(s\right)=k_{p2}=\frac{Torque}{e_{\psi }}=\frac{4.2}{0.102}=41.18,$$

(17) $$C\left(s\right)=k_{p3}=\frac{Torque}{e_{\psi }}=\frac{16.8}{0.102}=164.7.$$

**Figure 18:**
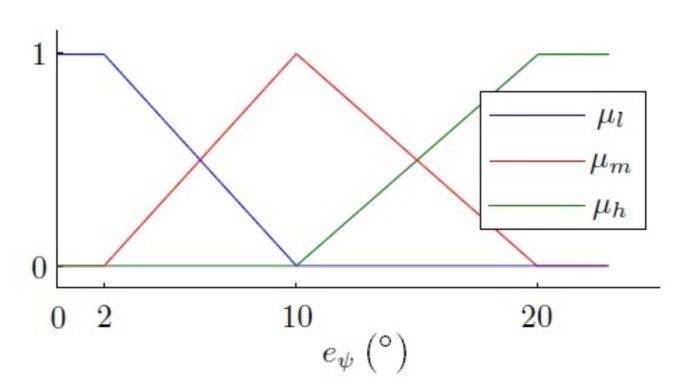
Yaw error fuzzy set. Membership functions $\mu_{l}$, $\mu_{m}$ and $\mu_{h}$.

The fuzzy control described above was used to choose the correct control for each scenario; the design aspects were not described. However, here, the fuzzy control is used to interpret the desired value, and so, the design aspects selected for the fuzzification and inference methods were: defining ‘low’, ‘medium’ and ‘high’ error as linguistic terms to control the yaw in a fuzzy way, for $2^{\circ }$, $10^{\circ }$ and $20^{\circ }$, obtaining the fuzzy set as follows, using triangular memberships,

(18) $$\mu_{l}\left(e_{\psi }\right)=\left\{\begin{array}{cc} 1 & \;\mathrm{if}\;\;e_{\psi }<2^{\circ }\\ \frac{10-e_{\psi }}{8} & \;\mathrm{if}\;\;2^{\circ }\leq e_{\psi }\geq 10^{\circ }\\ 0 & \;\mathrm{if}\;\;e_{\psi }>10^{\circ } \end{array}\right.,$$

(19) $$\mu_{m}\left(e_{\psi }\right)=\left\{\begin{array}{cc} 0 & \;\mathrm{if}\;\;e_{\psi }<2^{\circ }\\ \frac{e_{\psi }-2}{8} & \;\mathrm{if}\;\;2^{\circ }\leq e_{\psi }\geq 10^{\circ }\\ \frac{20-e_{\psi }}{10} & \;\mathrm{if}\;\;10^{\circ }\leq e_{\psi }\geq 20^{\circ }\\ 0 & \;\mathrm{if}\;\;e_{\psi }>20^{\circ } \end{array}\right.,$$

(20) $$\mu_{h}\left(e_{\psi }\right)=\left\{\begin{array}{cc} 0 & \;\mathrm{if}\;\;e_{\psi }<2^{\circ }\\ \frac{e_{\psi }-10}{10} & \;\mathrm{if}\;\;2^{\circ }\leq e_{\psi }\geq 10^{\circ }\\ 1 & \;\mathrm{if}\;\;e_{\psi }>10^{\circ } \end{array}\right.,$$

which can be graphically represented as is shown in Figure 18.

The linguistic rules to control the yaw using this error as input are:

(21) $$\begin{array}{cc} R_{1}: & \;\mathrm{if}\;|e_{\psi }|\;\mathrm{is}\;\mu_{l}\;\mathrm{then}\;C\left(s\right)\;\mathrm{is}\;k_{p1},\\ R_{2}: & \;\mathrm{if}\;|e_{\psi }|\;\mathrm{is}\;\mu_{m}\;\mathrm{then}\;C\left(s\right)\;\mathrm{is}\;k_{p2},\\ R_{3}: & \;\mathrm{if}\;|e_{\psi }|\;\mathrm{is}\;\mu_{h}\;\mathrm{then}\;C\left(s\right)\;\mathrm{is}\;k_{p3}. \end{array}$$

For the output, a Type-1 TSK fuzzy controller is used, which uses crisp functions instead of linguistic terms. To use it, a different weight for each yaw error has been calculated, obtaining the following equations:

(22) $$\begin{array}{c} \alpha_{\psi 1}=\mu_{l}\left(e_{\psi }\right),\\ \alpha_{\psi 2}=\mu_{m}\left(e_{\psi }\right),\\ \alpha_{\psi 3}=\mu_{h}\left(e_{\psi }\right), \end{array}$$

(23) $$\gamma_{\psi }=\frac{\alpha_{\psi 1}k_{p1}+\alpha_{\psi 2}k_{p2}+\alpha_{\psi 3}k_{p3}}{\alpha_{\psi 1}+\alpha_{\psi 2}+\alpha_{\psi 3}}.$$

### 3.3. Outer Loop

In the above section, different controls for the inner loop have been presented, which control the forward velocity and yaw of the vehicle. In this section, the pure pursuit approaches to design the outer loop are presented.

#### Pure Pursuit

**Figure 19:**
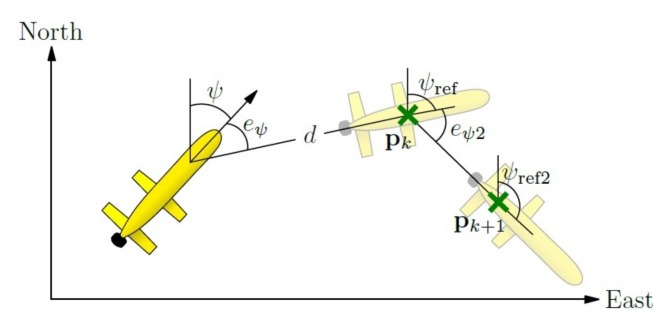
Pure pursuit: fuzzy control.

The design of Guanay II implies that the turn velocity is highly dependent on the forward velocity because if the vehicle moves at a high speed, the power to turn is low, but high if the velocity is low. Therefore, in this section, a method using fuzzy controllers is proposed, which will reduce the velocity of the Guanay II with respect to three parameters: the yaw error $e_{\psi }$, the distance to the waypoint *d* and the angle that the vehicle has to point to after it reaches the waypoint $\psi_{ref2}$; this idea is shown in Figure 19.

The yaw reference is calculated through the current position of the vehicle $p={\left[x,y\right]}^{T}$ and the position of the waypoint to reach, denoted as $p_{k}$, as:

(24) $$\psi_{ref}=\tan^{-1}\left(\frac{y_{k}-y}{x_{k}-x}\right),$$

and the yaw reference after reaching the waypoint is:

(25) $$\psi_{ref2}=\tan^{-1}\left(p_{k+1}-p_{k}\right)=\tan^{-1}\left(\frac{y_{k+1}-y_{k}}{x_{k+1}-x_{k}}\right),$$

which yield a yaw error after reaching the waypoint equal to:

(26) $$e_{\psi 2}=\psi_{ref2}-\psi_{ref}.$$

On the other hand, the distance between the position of the vehicle and the waypoint can be calculated as:

(27) $$d=\sqrt{e_{xy}^{T}\cdot e_{xy}}=\sqrt{{\left(x_{k}-x\right)}^{2}+{\left(y_{k}-y\right)}^{2}}.$$

**Figure 20:**
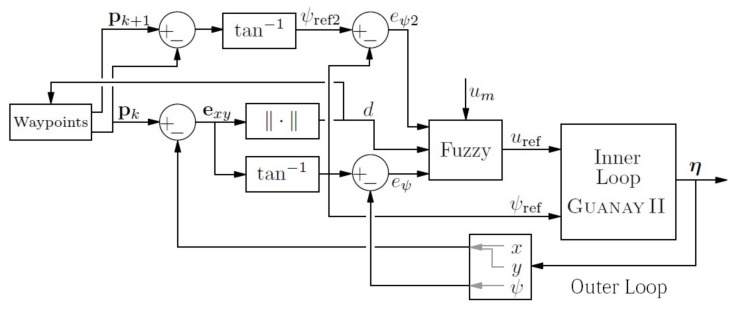
Outer loop: pure pursuit using a fuzzy controller.

This control is represented in the block diagram, which is shown in Figure 20.

**Figure 21:**
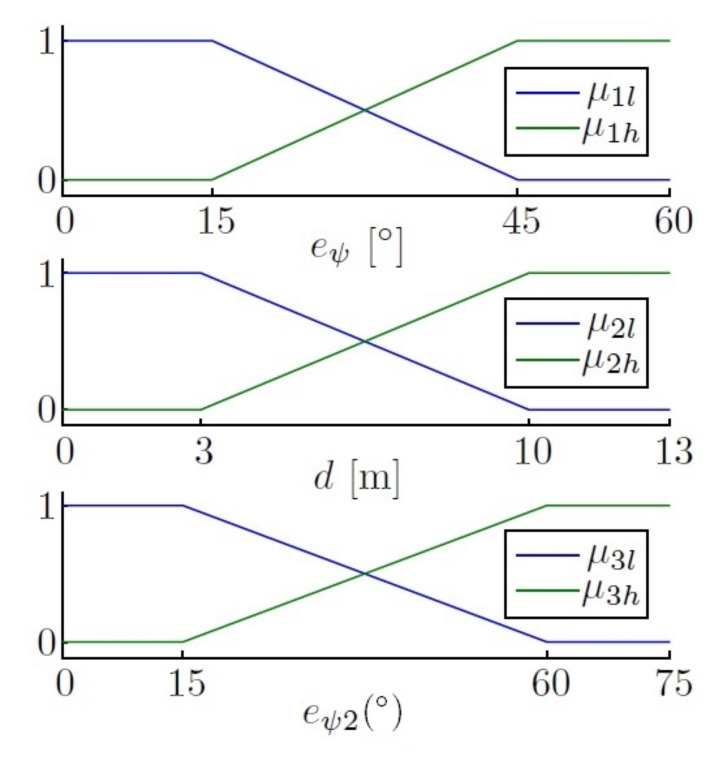
Membership functions of the fuzzy set of the outer loop.

Finally, the calculation of $u_{ref}$ is defined using a Type-1 TSK fuzzy controller, which has the following values and membership functions used for the fuzzification, represented in Figure 21. The rules are composed using a combination of these variables and summarized in Table 1, where $u_{m}$ is the velocity of the mission.

These rules give us the following weights:

(28) $$\begin{array}{cc} \alpha_{1}= & \min\{\mu_{1}l\left(e_{\psi }\right),\mu_{2}l\left(e_{d}\right),\mu_{3}l\left(e_{\psi 2}\right)\}\\ \alpha_{2}= & \min\{\mu_{1}l\left(e_{\psi }\right),\mu_{2}l\left(e_{d}\right),\mu_{3}l\left(e_{\psi 2}\right)\}\\ \alpha_{3}= & \min\{\mu_{1}h\left(e_{\psi }\right),\mu_{2}l\left(e_{d}\right)\}\\ \alpha_{4}= & \min\{\mu_{1}l\left(e_{\psi }\right),\mu_{2}h\left(e_{d}\right)\}\\ \alpha_{5}= & \min\{\mu_{1}h\left(e_{\psi }\right),\mu_{2}h\left(e_{d}\right)\}. \end{array}$$

Finally, using the crisp function of the TSK controller, the velocity reference is:

(29) $$u_{ref}=\frac{0.8\alpha_{1}+0.2\alpha_{2}+\alpha_{4}+0.3\alpha_{5}}{\sum_{m=1}^{5}\alpha_{m}}u_{m}.$$

## 4. Results

First of all, the results obtained with the vertical navigation and propulsion system explained in Section 2 are presented. Finally, the results of the automatic navigation control in the horizontal plane are presented, which is explained in Section 3. In both cases, simulations and field tests have been carried out.

### 4.1. Vertical Navigation Results

**Figure 22:**
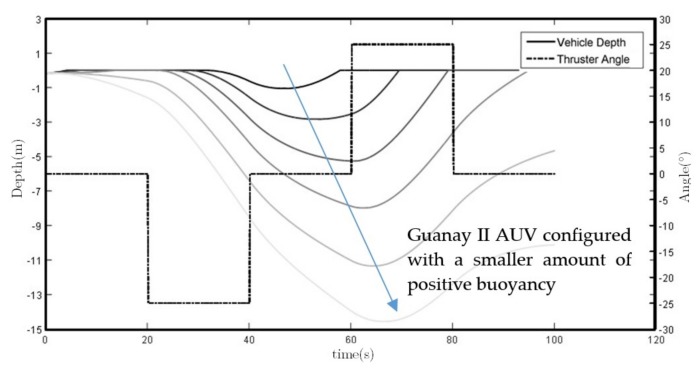
The vehicle’s vertical plane trajectory simulation with different buoyancy configurations, using the lateral thrusters at +25 and −25 degrees of inclination. This simulation shows that if the vehicle has a low buoyancy, the thrusters may not have enough force to bring the vehicle back to the surface. Therefore, careful buoyancy adjustment is mandatory before each mission.

With the new thruster vector control system designed, Guanay II has now full maneuverability, which allows us to control the vehicle in the horizontal and vertical plane. Its performance was simulated using the vehicle’s mathematical model explained above (see Equations (1) and (3)), whose values for the vertical plane obtained the results presented in Figure 22. In this simulation, the strong influence that the initial buoyancy has on the system is shown, whereas with high positive buoyancy, the thrusters cannot submerge the vehicle; the vehicle cannot be brought back to the surface without a positive buoyancy.

**Figure 23:**
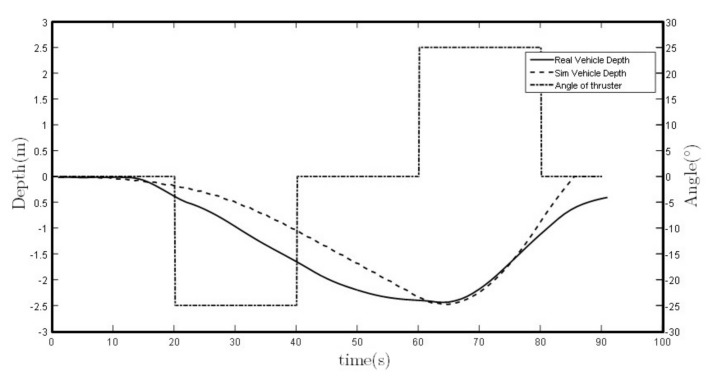
Vehicle’s vertical plane trajectory performance comparison between simulation and field test.

Finally, field tests have been conducted to compare and validate the simulations. Different immersions with different types of buoyancy levels, thruster force and thruster angles have been used to observe its performance, validating this method to control the depth of the Guanay II vehicle during an immersion trajectory. Figure 23 shows a comparison between a simulation and a real vehicle performance as an example.

Some small deviations between the simulation and the field test are observed. These can be caused by a difference between the adjustment of the model coefficients and the final configuration of the AUV, such as its buoyancy position or the real inclination of the lateral thrusters during the test.

### 4.2. Horizontal Navigation Results

Here, the final results of both inner and outer loop controllers are presented.

#### 4.2.1. Inner Loop Simulations Using the Fuzzy Controller

**Figure 24:**
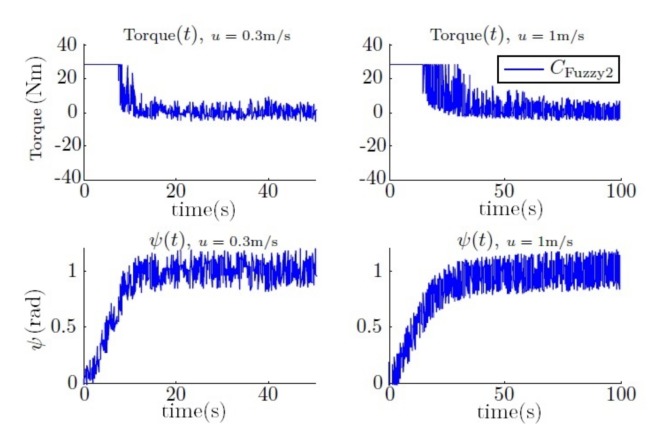
Step response of the yaw and torque using the fuzzy controller.

After different simulations, it is concluded that a large gain helps to achieve a specific angle faster, but in contrast, a low gain helps to reduce the switching torque on the thrusters. Therefore, the fuzzy controller is presented as an interpolation between them regarding the yaw error. The simulation result can be observed in Figure 24, where its performance can be observed, and compared with the results presented in the previous section (see Figure 15).

Finally, Table 2 resumes the performance of $C_{Fuzzy2}$ compared to the other proportional controllers.

#### 4.2.2. Outer Loop Simulations Using Fuzzy Controllers

For these simulations, three mission velocities have been chosen, 0.3 m/s, 0.6 m/s and 1 m/s. For the inner loop, $C_{Fuzzy1}^{\left(u\right)}$ and $C_{Fuzzy2}$ have been used to control the velocity and yaw, respectively.

**Figure 25:**
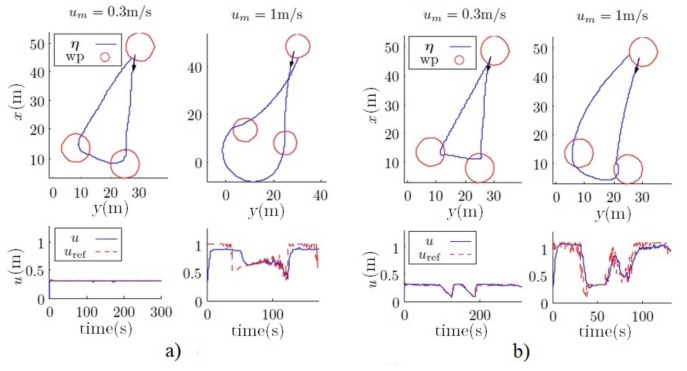
(**a**) Responses using the pure pursuit regarding the radius of curvature. (**b**) Responses using pure pursuit with the fuzzy controller.

Figure 25 shows the response of the vehicle and the way in which the fuzzy controller reduces the velocity of the vehicle when it is near a waypoint, maintaining a good performance for all the velocities. This performance can be shown especially at higher velocities.

#### 4.2.3. Field Tests

To validate the results obtained through simulations, different field tests have been carried out on both open sea around the OBSEA underwater cabled observatory area (www.obsea.es) [43], as well as in calm waters. The results presented here were taken in the Olympic Canal of Catalonia, which is both very calm and large. For these simulations, the $C_{Fuzzy1}^{\left(u\right)}$ controls have been used in the inner loop to control the velocity and the pure pursuit with a fuzzy control as the outer loop.

**Figure 26:**
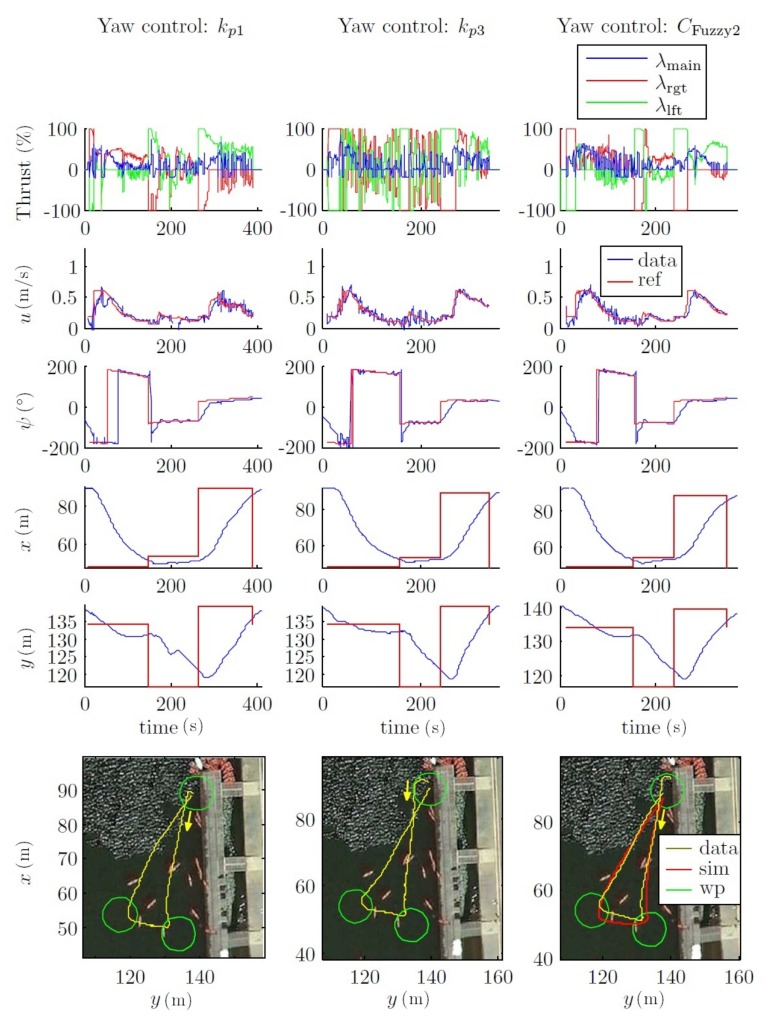
Field tests. Outer loop fuzzy control. Comparison of $C_{Fuzzy2}$ with proportional controllers. Path 1. *u* = 0.6 m/s. Where data is the real data acquired during the test, sim is the simulation results, and wp are the way-points to reach.

For example, Figure 26 shows a field test with a mission velocity equal to 0.6 m/s, using three types of controller to control the yaw: two proportional controllers, $k_{p1}$ and $k_{p3}$; and the fuzzy control $C_{Fuzzy2}$. For both $k_{p1}$ and $C_{Fuzzy2}$, the thrusters were not saturated by the control action. Moreover, $C_{Fuzzy2}$ achieves the yaw reference more quickly than the other controllers.

**Figure 27:**
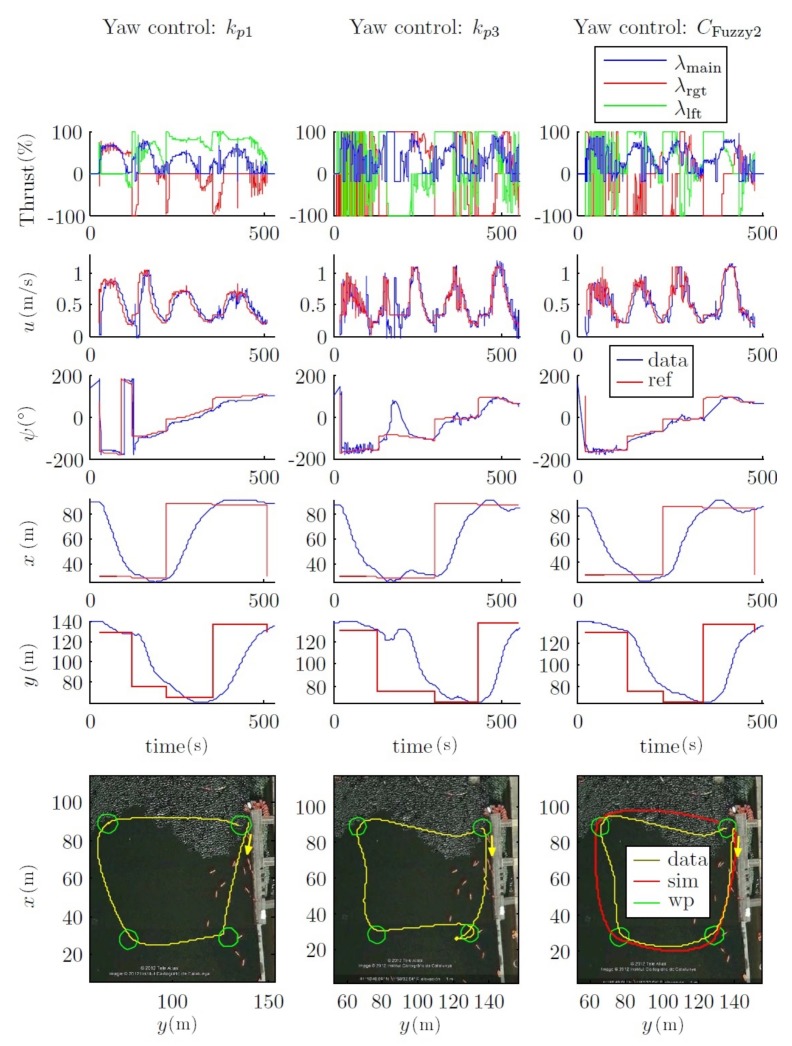
Field tests. Outer loop fuzzy control. Comparison of $C_{Fuzzy2}$ with proportional controllers. Path 2. *u* = 1 m/s.

This dynamic could also be observed in Figure 27, where the path of the field test was designed with more distance between waypoints and with a mission velocity equal to 1 m/s. This can be observed on XY and the yaw chart.

## 5. Discussion

Increasingly, robotic solutions replace routine jobs on land, in space and in the sea, in industry [44]. In particular, due to the high operational costs and the limited human accessibility to the marine environment, the potential of autonomous robotic actions is even higher than on land. The aim of this paper was to study and develop a new robotized vehicle as a platform in support of applications in marine, geosciences, ecology and archeology, which have been increasingly relying on mechatronic solutions for at-sea operations in the past 30 years [5]. Here, innovations at the level of hardware and software have been established, to potentiate AUV autonomous operability, by adding novel mechatronic insight to across-depth navigation and trajectory control. The study, implementation and then testing of a specific AUV configuration in a real environment have been carried out, which include, but are not limited to, the installation of thrusters. At the same time, the control issues of these kinds of vehicles have been addressed, where comparisons between different navigation systems were carried out through both simulations and field tests. From a control systems design point of view, this work advanced the use of methods for motion control that rely heavily on fuzzy techniques. Taken together, those advancements would contribute to expanding the use of versatile AUV platforms within the framework of fast growing permanent marine ecological monitoring networks, combining fixed and mobile robotic platform designs, which are being deployed to monitor ecologically- or industrial-extractive relevant continental margin and abyssal areas [45]. In this context, our solutions propose a step forward toward AUV autonomy that will eventually lead to an in situ docking at pelagic or benthic fixed nodes.

Vectorial propulsion systems are widely used, especially in Remotely-Operated Vehicles (ROVs). However, in AUVs, those methods are comparatively less implemented. In our study, a clear example for that situation has been described, since our vehicle was potentiated with thruster solutions, which were not commercially available at the standard level. Whereas vectorial propulsion systems are not new (see some other solutions as examples in [46,47]), each vehicle has its own design and characteristic constraints, which have to be carefully taken into consideration prior to customization planning. Therefore, low-cost, off-the-shelf components that had to be adapted to our design have been bought; i.e., to obtain a thruster vector control on the vertical plane, which allows us to adjust the angle between the lateral thrusters and the hull through actuators installed on the rear fins.

From the point of view of the control systems design, the paper presents novel advancements about methods for motion that chiefly rely on fuzzy control techniques through simulations, but also field tests, as a main difference with respect to previously published papers, e.g., [30]. This work clearly showed how the developed algorithms were efficient in enhancing the motion control capabilities. Whereas the navigation control strategy used an already known approach (i.e., the pure-pursuit; see as an example [48]), the implemented methodology through diffuse techniques is entirely new and specifically described in our script.

Most of the works about control systems implement the controllers using the vehicle hydrodynamic model at specific forward velocity to simplify the design [21,22,23]. Whereas this is correct for vehicles that usually navigate in open seas, where velocity is mainly constant, this method should not be used in other scenarios, such as in the interior of harbors and canals. In these situations, the variation of the forward velocity becomes relevant, and it is then important to be able to vary the controller’s working point to adjust the paths to the desired ones. Some authors have designed a two-step control approach [49], which switches between two controls designed for ‘high’ and ‘low’ velocities. However, the decision to change between one controller to the other one is not trivial. To solve this problem, Silvestre and Pascoal [26] use a set of linear controllers adjusted for different forward velocities and then use a gain scheduling controller to integrate them. Here, the same methodology has been followed, but innovatively applying a fuzzy controller, to integrate the different linear controllers. The fuzzy controller allows activation zones to be established, which can be controlled through fuzzy sets. This method showed good navigation performance and was used as interpolation between different rules or linear controls. This approach has also been used recently in other papers (e.g., [30]), where the navigation performance was simulated with 6 DOF.

Here, two controllers have been designed to provide autonomous navigation capabilities: one for the inner loop (dynamic), which is in charge of controlling the thrusters to reach a reference yaw and forward velocity; the other for the outer loop (kinematic), which is in charge of generating the yaw and forward velocity references, according to the waypoints and the vehicle’s current state. With respect to the inner loop, two solutions have been presented for velocity and yaw control based on Type-1 TSK fuzzy controller. These controllers were used to manage, at a higher level, different linear controllers designed for specific scenarios, such as different forward velocities. The inner loop developed to control the vehicle’s velocity and yaw results from a vehicle’s linear model in sections obtained from its non-linear model, where the vehicle’s structural characteristics have to be taken into account. When linear controllers (i.e., PID) are used, diffuse techniques were implemented to provide the adaptive navigation capability. On the other hand, the detailed study, development and identification of a dynamic model were required to take into account the hydrodynamic effects and propeller characteristics, which have also been presented.

With respect to the outer loop, we have presented a solution for pure pursuit navigation, where the radius of curvature of the vehicle is taken into account while trying at the same time to preserve the forward velocity, using also a Type-1 TSK fuzzy controller. The main advantage of this class of non-linear controllers, in front of others such as gain-scheduled ones [26,50], is that it is based on the zonal capacity of the linguistics law. This allows us to adjust the theoretical laws into specific zones and interpret those laws as a function of their different zones. This performance is in contrast to the classic or digital logic one , which operates with discrete values. Moreover, this kind of zonal controller will allow us to include future laws on the vertical plane as an extra zone of functionality.

## 6. Conclusions

This paper presents a new thruster vector control system, which allows depth navigation control. This system was implemented on Guanay II AUV and has been evaluated and tested through different simulations and field tests, which demonstrate the performance of this system and its capability to be used as a vectorial navigation system.

Moreover, a complete study on automatic navigation control has been presented, where two fuzzy controllers have been developed to solve non-linear properties in both inner and outer loop controls; for example, the presence of noise on the yaw measurements introducing fast switching into the thrusters’s control and a fuzzy control based on distance and angle error to the next waypoints to control the vehicle’s velocity, which yields greater accuracy on the trajectory of the vehicle.

All these considerations are shown in the field tests, where two comparative mission velocity (i.e., 0.6 m/s and 1 m/s; see Figure 26 and Figure 27) types of trajectories are represented. In both cases, the forced turn performances (to go to the next waypoint) were greatly increased. This occurred because the forward velocity was reduced when the vehicle reached a waypoint. With respect to the power used by the thrusters, it can be observed that both the $k_{p1}$ and $C_{Fuzzy2}$ controls did not saturate the thrusters.

Moreover, the simulated trajectory using $C_{Fuzzy2}$ was very similar to the experimental one. However, the trajectories south-north and west-east had a small deviation during the $k_{p3}$ and the $C_{Fuzzy2}$ tests (see Figure 27), probably due to a compass misalignment or to the increase of the sea currents in the test canal because of changes in the weather conditions.

On the other hand, the depth navigation performance is shown in Figure 22 and Figure 23, where the influence of the vehicle buoyancy is observed, which should be taken carefully into consideration before each mission.

Finally, a controller method for the vertical plane, and its modeling in conjunction with the horizontal plane, to allow more complex trajectories in 3D, should be addressed as future work, as well as the implementation of other controller techniques, such as path following, which would allow the vehicle to follow a specific path instead of simple waypoints.

## Figures and Tables

**Table 1:** The control rules for the velocity reference.

Rule	$|e_{\psi }|$	*d*	$|e_{\psi 2}|$	$u_{\mathrm{ref}}$
$R_{1}$	small	small	small	$0.8u_{m}$
$R_{2}$	small	small	big	$0.2u_{m}$
$R_{3}$	big	small	-	0
$R_{4}$	small	big	-	$u_{m}$
$R_{5}$	big	big	-	$0.3u_{m}$

**Table 2:** Comparison of the settling time and noise in the torque using the fuzzy controller and proportional controllers.

Controller	Settling Time (s)		STD in Torque (Nm)	
	0.3 m/s	1 m/s	0.3 m/s	0.3 m/s
$k_{p1}$	29.9	98.0	2.9	3.0
$k_{p2}$	19.3	65.3	4.3	4.1
$k_{p3}$	11.1	30.3	16.4	14.5
$C_{Fuzzy2}$	12.2	47.5	3.6	4.9
